# Supplementary material for: Content-rich biological network constructed by mining PubMed abstracts
Source: BMC Bioinformatics. 2004 Oct 8;5:147. doi: 10.1186/1471-2105-5-147 (PMC528731; doi:10.1186/1471-2105-5-147)
Supplement: Additional File 2 — The original results of the above study (non-essential files are deleted to keep the file size under the limit set by BMC bioinformatics). [file 1471-2105-5-147-S2.bz2 › chilibotAdditionalFile2/dip05/603ID9751050E10982/html/CDK6_CDKN2D.html]

 


 **CDK6** and **CDKN2D** 
  
Found 24 abstracts in PubMed, retrieved 05.  
 

 What does Google say? 
 PDF only 
| .edu only 

---

**Interactive relationship** (e.g. stimulation, inhibition, etc)

**Inhibitory relationship**- While members of the INK4 family p16INK4A, p15INK4B, p18INK4C, p19INK4D  [ **CDKN2D** ]  interact specifically with CDK4 and  **CDK6** , the CIP KIP inhibitors p21CIP1 WAF1, p27KIP1 and p57KIP2 inhibit a broader spectrum of CDK.  Ref: 11355576 Cancer Radiother, 2001
- We also review our recent structural studies on intracellular signalling complexes, focusing on phosducin transducin GPry, CK2 protein kinase and its complexes, and the cyclin D dependent kinase,  **Cdk6** , bound to the cell cycle inhibitor p19INK4d  [ **CDKN2D** ] .  Ref: 11076027 Biol Chem< MedlineTA>Biol Chem, 2001
**Stimulatory relationship**- The kinase activation was found to result from Tax induced expression of genes for cell cycle regulatory molecules including cyclin D2, cyclin E, E2F1, CDK2, CDK4 and  **CDK6** , and Tax induced reduction of CDK inhibitors p19 INK4d  [ **CDKN2D** ]  and p27 Kip1.  Ref: 11360190 Oncogene, 2001
- P19 INK4d  [ **CDKN2D** ]  is a tumor suppressing protein and belongs to a family of cyclin D dependent kinase inhibitors of CDK4 and  **CDK6** , which play a key role in human cell cycle control.  Ref: 11786024 J Mol Biol, 2002
**Neutral relationship**

**Non-interactive relationship** (e.g. studied together, co-existance, homology, etc.)

- Analysis of normal B cell precursors by Western blotting indicated that CDK4,  **CDK6** , p19 INK4d  [ **CDKN2D** ] , and p57 KIP2 were expressed.  Ref: 11301189 Exp Hematol, 2001
